# Loose and Tight GNSS/INS Integrations: Comparison of Performance Assessed in Real Urban Scenarios

**DOI:** 10.3390/s17020255

**Published:** 2017-01-29

**Authors:** Gianluca Falco, Marco Pini, Gianluca Marucco

**Affiliations:** Istituto Superiore Mario Boella, Torino 10138, Italy; pini@ismb.it (M.P.); marucco@ismb.it (G.M.)

**Keywords:** GNSS/INS integration, horizontal positioning errors, urban navigation

## Abstract

Global Navigation Satellite Systems (GNSSs) remain the principal mean of positioning in many applications and systems, but in several types of environment, the performance of standalone receivers is degraded. Although many works show the benefits of the integration between GNSS and Inertial Navigation Systems (INSs), tightly-coupled architectures are mainly implemented in professional devices and are based on high-grade Inertial Measurement Units (IMUs). This paper investigates the performance improvements enabled by the tight integration, using low-cost sensors and a mass-market GNSS receiver. Performance is assessed through a series of tests carried out in real urban scenarios and is compared against commercial modules, operating in standalone mode or featuring loosely-coupled integrations. The paper describes the developed tight-integration algorithms with a terse mathematical model and assesses their efficacy from a practical perspective.

## 1. Introduction

During the last years, there has been an increasing demand for accurate estimate of users’ position in many systems and applications, such as driving assistance systems and autonomous vehicles. In addition to enhanced performance in different types of operational environments, developers seek innovative strategies for reliable systems at affordable costs [1,2,3]. Unfortunately, the urban environment poses some of the most severe challenges to Global Navigation Satellite Systems (GNSSs) that remain the principal mean of positioning for outdoor navigation. Indeed, the presence of buildings and trees induce signal reflections and attenuations that in turn provoke measurements affected by errors. In severe cases, the number of visible satellites is not sufficient and receivers are unable to provide Position Velocity and Time (PVT) data.

Although multiple constellations improve the satellites’ visibility [4], the performance of standalone receivers in urban settings can be enhanced following two main strategies. First, receivers can be augmented with sensors, such as wheel odometers [5], Inertial Navigation Systems (INSs) [6,7,8], Light Detection and Ranging (LIDAR) [9], or can be combined with other terrestrial systems, such as Wi-Fi networks. Second, the position accuracy of standalone receivers can be improved with innovative signal processing, such as high sensitivity tracking loops [10], Cooperative Positioning [11], or 3-Dimensional (3-D) building models to predict satellite visibility, as proposed in [12,13].

In this paper, we assess the performance of two well-known algorithms employed to integrate GNSS receivers and INSs. They represent two of the most common sensors that are typically used in a wide range of applications. Small, robust and low-cost inertial sensors (e.g., Micro Electrical Mechanical Sensors (MEMS) [7]) have been available on the market for several years and are combined with Global Positioning System (GPS) receivers especially for land vehicle navigation. During the last decade, different approaches for GPS/INS integration have been adopted [14] and many of them have been investigated for different grades of Inertial Measurement Units (IMUs). The three most common integration strategies are: the loosely-coupled [15], the tightly-coupled [16] and the ultra-tight integration [17,18]. Since the ultra-tight integration involves the baseband signal processing of GNSS receivers (i.e., the digital tracking loops), that is typically not accessible using commercial products, this paper compares loosely and tightly-coupled techniques employing Commercial Off The Shelf (COTS) modules. Briefly, the basic difference between them is the type of data shared by the GPS receiver and the INS sensors. In the loosely-coupled technique, the positions and velocities estimated by the GPS receiver are blended with the INS navigation solution, while in the case of tightly-coupled method, GPS raw measurements (i.e., pseudorange and Doppler observables) are processed through a unique Kalman filter with the measurements coming from the inertial sensors to estimate the PVT. The main advantage of the tight integration [16] is the possibility to update the hybrid navigation solution also in scenarios with poor signal quality or limited coverage, thanks to the prediction of pseudoranges and Doppler trends.

Many papers highlight the benefits of tightly-coupled architectures in urban environments. For example, in [3] authors developed a tight method with additional constraints on velocity and height to maintain the INS errors bounded in case of GPS outage. In [19], an algorithm able to reduce the effect of multipath and monitor the quality of the pseudoranges has been implemented within a tight architecture. Similarly, in [10], a correction is applied to the received GPS signals to smooth the errors affecting the pseudoranges. In [20], authors investigated the combined use of tightly-coupled integration and an off-line Kalman filter to provide a high accuracy position and attitude solution for urban environment. Eventually, reference [21] analyzes the advantages of a tightly-coupled algorithm as a position, velocity, and attitude estimator for autonomous navigation. A statistical sensitivity analysis was performed by considering the effects of map aiding, differential corrections and carrier phases. Even if the tightly-coupled method has shown remarkable advantages in harsh environments, such architecture is still a research topic [16,17,18,19,20,21,22] or is mainly implemented only for professional applications and based on high-grade IMUs. In fact, most of MEMS IMUs [23,24] continue to be integrated with GNSS receivers, through loosely-coupled schemes [23]. However, in the years ahead, an improvement of the performance of MEMS IMUs that will enable low-cost positioning systems based on tightly-coupled architecture is expected.

In this paper, we investigate the performance improvements enabled by the tight integration, using low-cost sensors. Performance is assessed in a real urban context and is compared against those obtained by a commercial MEMS IMU, loosely integrated with a mass-market GPS receiver.

Although many papers describe loosely-coupled integrations for automotive applications [20,25,26], limited work [27] has been done to assess the efficacy of tight navigation solutions, based on low-cost devices, from a practical perspective. Most of the papers compare the loosely-coupled system performance only with respect to a tight solution obtained through a highly precise GPS Real Time Kinematic (RTK) with an accurate tactical grade IMU [19]. Other papers estimate the positional solution accuracy obtained through a loosely-coupled and tightly-coupled architecture exclusively via simulation by reducing off-line the number of satellites in visibility [15,25].

Therefore, in many examples available in literature, there is no fair comparison of the “figures of merit.” The paper provides a detailed description of the main features of the tightly-coupled algorithm that has been designed on top of a mass-market, single-frequency GPS receiver and a low-cost INS. Then, the paper describes the results of some tests performed in three urban scenarios: the first in open sky conditions the second driving through narrow streets in downtown Turin, the third along a straight avenue of trees.

More specifically, the paper is organized as follows: after this introduction, the first section briefly describes the architecture of a loosely-coupled GPS/INS system and the main features of the designed tightly-coupled algorithm. The following section presents the scenarios where we run the test campaign and the metrics used to assess the performance of the algorithms under investigation. In the last section, the results are shown and thoroughly discussed.

## 2. Navigation Algorithms Based on Global Navigation Satellite System (GNSS)/Inertial Navigation Systems (INS) Integrations

In this section, we briefly recall the architecture of a loose integration that is embedded within many commercial devices, including the commercial MEMS IMU used for comparison in the test campaign. The section also provides mathematical details about the design of the tightly-coupled algorithm as well as some insights on its real-time implementation on an embedded system.

### 2.1. Common Loosely-Coupled Architecture

**Figure 1:**
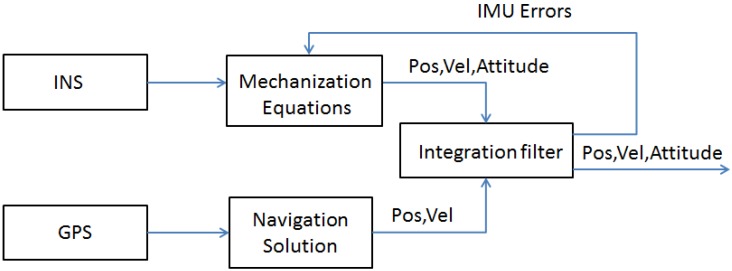
Block diagram of a common loosely-coupled architecture.

The most common GNSS/INS integration scheme is the so-called loosely-coupled where the positions and velocities derived by GNSS signal processing are merged as updates of the INS estimates positional information, through a navigation Kalman filter [7]. To further improve the accuracy of the navigation solution, the error states are fed back to the INS mechanization equation [6] to mitigate the errors that affect the IMU [3]. A simple block diagram of loosely-coupled architecture is reported in Figure 1.

The INS mechanization equations are used to convert the inertial measures (accelerations and angular rates along the three orthogonal directions) from the body frame (*b*-fame) to the navigation frame (e.g., the local *l*-frame or the ECEF-frame) and they are omitted in the following of the paper. A detailed description of such equations can be found in [7] for the *l*-frame and in [16] for the ECEF-frame transformations, respectively. Considering an ECEF-frame, the typical error states estimated by the GPS/INS integration Kalman filter are:

(1) $$\delta x=\left[\underset{\delta r}{\underset{︸}{\delta x\;\delta y\;\delta z}}\;\underset{\delta v}{\underset{︸}{\delta V_{x}\;\delta V_{y}\;\delta V_{z}}}\;\underset{\delta A}{\underset{︸}{\delta A_{x}\;\delta A_{y}\;\delta A_{z}}}\;\underset{\delta \omega }{\underset{︸}{\delta \omega_{x}\;\delta \omega_{y}\;\delta \omega_{z}}}\;\underset{\delta f}{\underset{︸}{\delta f_{x}\;\delta f_{y}\;\delta f_{z}}}\right]$$

where δr, δv, δA are the errors related to position, velocity and attitude while δω and δf are the errors associated with the IMU gyroscopes and accelerometers, respectively.

A generic measurement model of a discrete time Kalman filter can be written as stated in (2):

(2) $$z_{k}=H_{k}x_{k}+\nu_{k}$$

where $H_{k}$ is the matrix that shows the relationship between the error states and the measurements $z_{k}$ at the k-th time instant, whereas $\nu_{k}$ is the white noise associated with the measurements with a covariance matrix equal to $R_{k}$. In case of loosely-coupled expressed in ECEF coordinates, $z_{k}$ can be written as:

(3) $$z_{k}={\left[X_{\mathrm{INS}}-X_{\mathrm{GPS}},Y_{\mathrm{INS}}-Y_{\mathrm{GPS}},Z_{\mathrm{INS}}-Z_{\mathrm{GPS}}\right]}^{T}$$

A dynamic model of an error state discrete Kalman filter is:

(4) $$\delta x_{k}=\Phi_{k-1}\delta x_{k-1}+\mu_{k-1}$$

where Φ is the transition matrix that relates two consecutive error states vectors and μ indicates the noise propagation in the discrete time system. Details about the design of the matrix Φ can be found in [7].

### 2.2. Common Tightly-Coupled Architecture

**Figure 2:**
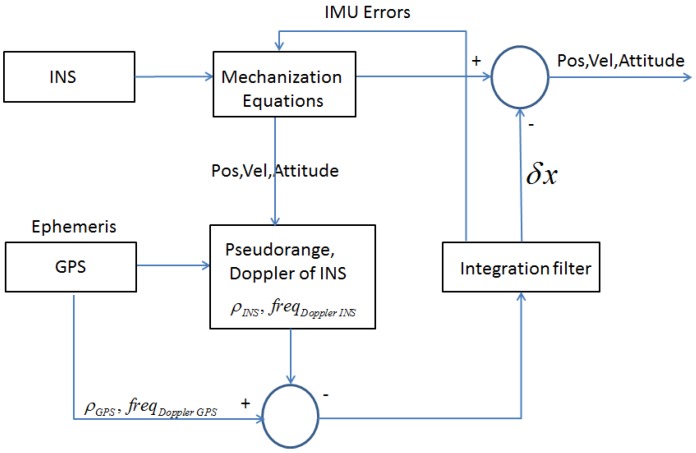
Block diagram of a common tightly-coupled architecture.

A tightly-coupled algorithm uses a centralized Kalman filter that integrates the estimated pseudoranges ($\rho_{\mathrm{GPS}}$) and Doppler shift ($freq_{\mathrm{Doppler},\mathrm{GPS}}$) from the GNSS receiver and the information of position, velocity and attitude coming from the mechanization equations of the inertial sensors. A simple block diagram of a tightly-coupled architecture is reported in Figure 2.

Considering an *ECEF*-frame navigation, the error states estimated by the GNSS/INS integration Kalman filter can be written as:

(5) $$\delta x=\left[\underset{\delta r}{\underset{︸}{\delta x\;\delta y\;\delta z}}\;\underset{\delta v}{\underset{︸}{\delta V_{x}\;\delta V_{y}\;\delta V_{z}}}\;\underset{\delta A}{\underset{︸}{\delta A_{x}\;\delta A_{y}\;\delta A_{z}}}\;\underset{\delta \omega }{\underset{︸}{\delta \omega_{x}\;\delta \omega_{y}\;\delta \omega_{z}}}\;\underset{\delta f}{\underset{︸}{\delta f_{x}\;\delta f_{y}\;\delta f_{z}}}\;\underset{\delta b}{\underset{︸}{\delta t\;\delta t^{\prime }}}\right]$$

where δb includes the clock bias and drift errors that affect the GNSS receiver. Also in the case of a tightly-coupled architecture, the centralized Kalman filter is formed by a dynamic error states model and a measurements model. Going into detail, the discrete transition matrix $\Phi_{k}$ expressed in ECEF-frame, can be written according to [16] as:

(6) $$\Phi_{k}=\left[\begin{array}{ccccccc} I_{3\times 3} & T_{k}\cdot I_{3\times 3} & 0_{3\times 3} & 0_{3\times 3} & 0_{3\times 3} & 0 & 0\\ N^{e} & I_{3\times 3}-2T_{k}\Omega_{ie}^{e} & -T_{k}F_{k} & 0_{3\times 3} & T_{k}C_{b,k3\times 3}^{e} & 0 & 0\\ 0_{3\times 3} & 0_{3\times 3} & I_{3\times 3}-T_{k}\Omega_{ie}^{e} & T_{k}C_{b,k}^{e} & 0_{3\times 3} & 0 & 0\\ 0_{3\times 3} & 0_{3\times 3} & 0_{3\times 3} & I_{3x3}+T_{k}D_{g} & 0_{3\times 3} & 0 & 0\\ 0_{3\times 3} & 0_{3\times 3} & 0_{3\times 3} & 0_{3\times 3} & I_{3\times 3}+T_{k}D_{a} & 0 & 0\\ 0_{1\times 3} & 0_{1\times 3} & 0_{1\times 3} & 0_{1\times 3} & 0_{1\times 3} & 0 & 1\\ 0_{1\times 3} & 0_{1\times 3} & 0_{1\times 3} & 0_{1\times 3} & 0_{1\times 3} & 0 & 0 \end{array}\right]$$

where:

- $N^{e}$ represents the tensor of gravity gradients;
- $\Omega_{ie}^{e}$ is the Earth rotation rate;
- $F_{k}$ is the skew-symmetric matrix of the accelerometers measured at time-*k*;
- $C_{b,k}^{e}$ is the Direct Cosine Matrix (DCM)computed from the body to the earth frame;
- $D_{a}$ and $D_{g}$ are the time-constant diagonal matrices that define a first-state Gauss-Markov model for the accelerometers and the gyroscopes, respectively;
- $T_{k}$ is the time sampling interval between two consecutive executions of the dynamic model of the Kalman filter.

In order to take into account how the noise affecting the INS sensors is distributed among the state vectors parameter, (4) can be expanded as:

(7) $$\delta x_{k}=\Phi_{k-1}\delta x_{k-1}+G_{k-1}\omega_{k-1}$$

where $G_{k-1}$ is the noise distribution matrix. A mathematical expression can be found in [7]. The definition of the model noise vector $\mu_{k}$ is reported in (8):

(8) $$\mu_{k}={\left[\mu_{t,k},{\left(\mu_{a,k}\right)}^{T},{\left(\mu_{g,k}\right)}^{T},\mu_{t^{\prime },k},{\left(\mu_{aa,k}\right)}^{T},{\left(\mu_{gg,k}\right)}^{T}\right]}^{T}\;\mu_{k}\in {ℜ}^{14,1}$$

where:

- $\mu_{t,k}$ and $\mu_{t^{\prime },k}$ are the clock error and the clock drift error noises at the discrete time *k*, respectively;
- $\mu_{a,k}$ and $\mu_{g,k}$ are the additive white noise components on the three accelerometers’ gyros outputs;
- $\mu_{aa,k}$ and $\mu_{gg,k}$ are the bias instabilities of the IMU accelerometers and gyros, respectively.

Thus, the covariance matrix Q of the noise components, as stated in (8), can be computed as a diagonal matrix as reported in [16]. A measure of the noise standard deviation related to the receiver clock bias can be found in [28], while stochastic noises that generate the instability of the INS sensors (i.e., $\mu_{aa,k}$ and $\mu_{gg,k}$) are typically modelled as a 1st order Gauss-Markov process.

The discrete form of covariance matrix can be obtained according to the following formula [3]:

(9) $$Q_{k}\approx \frac{1}{2}\left[\Phi_{k}G_{k}Q(t_{k})G_{k}^{T}+G_{k}Q(t_{k})G_{k}^{T}\Phi_{k}^{T}\right]\cdot \Delta t$$

As far as the measurements model of the tightly-coupled Kalman filter is concerned, according to (2), the observation vector $z_{k}$ is defined as:

(10) $$z_{k}=\zeta_{sat,k}-{\hat{\zeta }}_{k}$$

where:

- $\zeta_{sat,k}={\left[\rho_{k},\;\rho_{k}^{\prime }\right]}^{T}\;\in {ℜ}^{2N_{sat},1}$ is the vector of corrected pseudoranges $\rho_{k}$ and pseudorange rates $\rho_{k}^{\prime }$ at the time instant *k*;
- ${\hat{\zeta }}_{k}={\left[{\hat{\rho }}_{k}\;{{\hat{\rho }}^{\prime }}_{k}\right]}^{T}\;\in {ℜ}^{2N_{sat},1}$ represents the predicted pseudorange and pseudorange rate vector, computed from the current estimate of the target trajectory.

The observation matrix, indicated with $H_{k}$, is time varying in case of a tightly-coupled architecture and it depends on the number of satellites in visibility. Such a matrix can be written as:

(11) $$H_{k}=\left[\begin{array}{cccc} H_{\rho ,k} & 0_{Nsat\times 3} & 0_{Nsat\times 3} & 0_{Nsat\times 8}\\ 0_{Nsat\times 3} & H_{\rho ,k} & 0_{Nsat\times 3} & 0_{Nsat\times 8} \end{array}\right]\;\in {ℜ}^{2Nsat,17}$$

where $H_{\rho ,k}$ is the Jacobian matrix of the non-linear relationship between the user’s position and clock and the *N_sat_* pseudoranges $\rho_{1},\ldots ,\rho_{Nsat}$, respectively. It is possible to write $H_{\rho ,k}$ as:

(12) $$H_{\rho ,k}={\left[\frac{\partial h[n]}{\partial x^{\left(p\right)}}\right]}_{x=\overset{⌣}{x}}=\left[\begin{array}{cccc} \frac{\overset{⌣}{x}-x_{1}}{d_{1}} & \frac{\overset{⌣}{y}-y_{1}}{d_{1}} & \frac{\overset{⌣}{z}-z_{1}}{d_{1}} & -1\\ \frac{\overset{⌣}{x}-x_{2}}{d_{2}} & \frac{\overset{⌣}{y}-y_{2}}{d_{2}} & \frac{\overset{⌣}{z}-z_{2}}{d_{2}} & -1\\ \vdots & \vdots & \vdots & \vdots \end{array}\right]$$

where $d_{j}$ is the norm of the vector [$x-x_{j}$, $y-y_{j}$, $z-z_{j}$] and [$\overset{⌣}{x}$, $\overset{⌣}{y}$, $\overset{⌣}{z}$] are the estimated user’s position coordinates, whereas $x_{1\cdots N}$, $y_{1\cdots N}$, $z_{1\cdots N}$ represent the *N* satellites positions in the ECEF-frame.

### 2.3. Designed Tightly-Coupled Architecture

**Figure 3:**
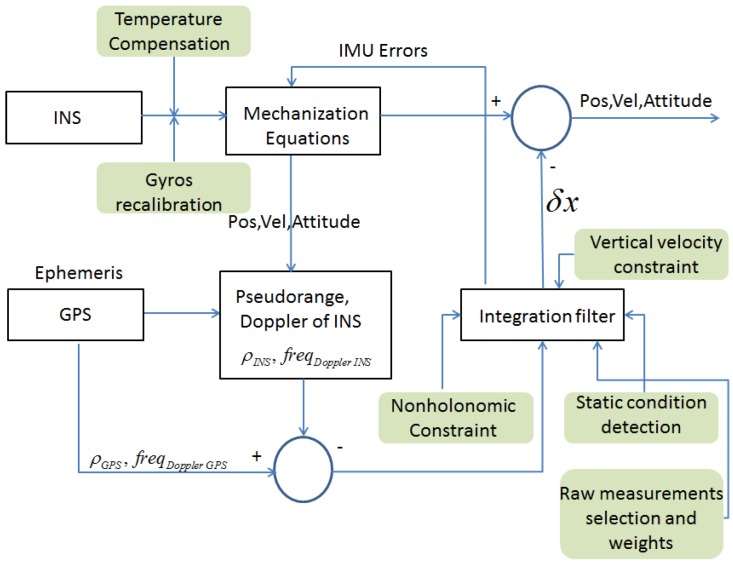
Block diagram of a designed tightly-coupled architecture.

With respect to the architecture described above, in our design we included additional constraints and features in order to improve the performance of the tightly-coupled algorithm, specifically for land applications. The block scheme of the new architecture is shown in Figure 3, where the additional functions are marked in green and detailed below.

#### 2.3.1. Temperature Compensation

**Figure 4:**
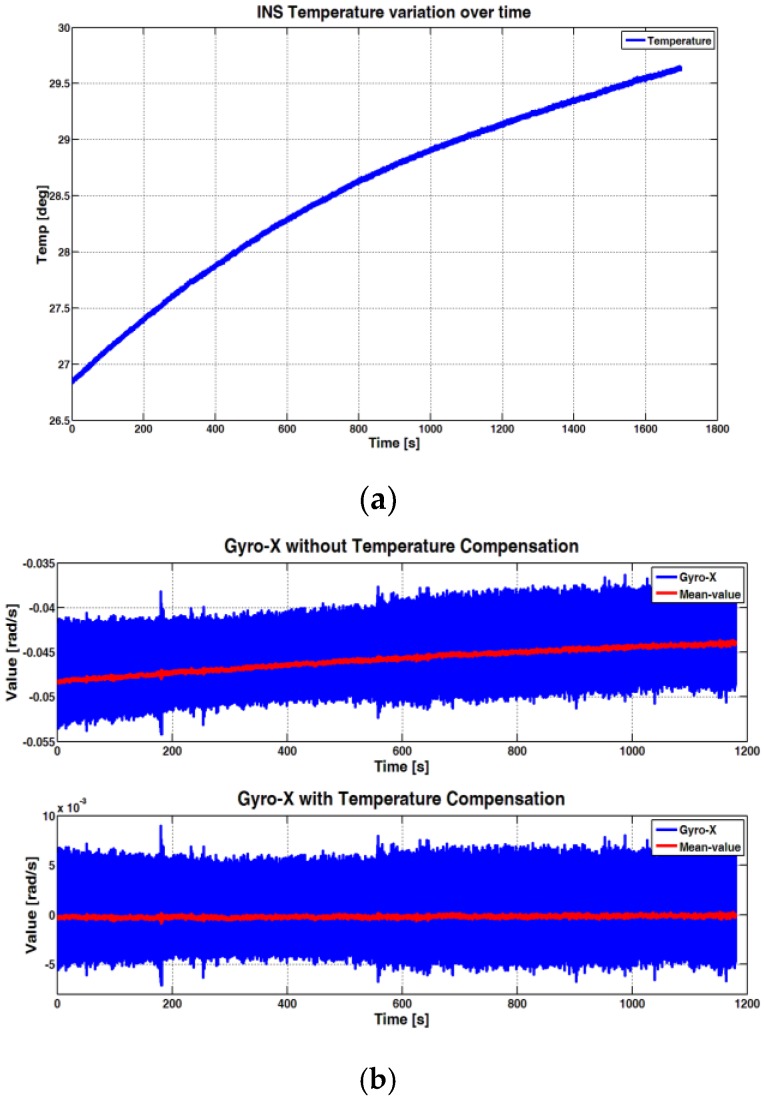
Temperature variation over time (**a**). Example of compensation for the Gyro-X compared with the same measurements without compensation (**b**).

IMUs based on gyros and accelerometers not compensated in temperature degrade the accuracy of the INS navigation solution. An example of a non-temperature compensated gyro is visible in Figure 4a, where the drift of the angular rate, according to the variation of the temperature over time in a static condition, is evident.

**Figure 5:**
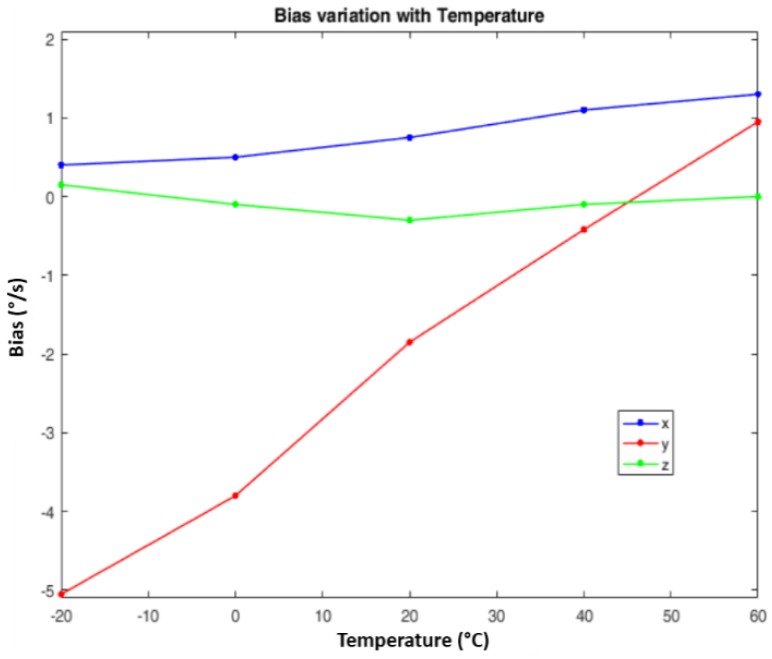
InvenSense MPU Gyros Bias vs. Temperature.

Most of the low-cost MEMS IMUs available on the market (e.g., [29]) are not temperature compensated. In our design, we estimated the INS sensors biases off-line, installing the IMU into a temperature-controlled chamber and rotating the IMU in different positions according to the characterization tests proposed in [30]. We repeated the same procedure for different temperatures in the range –20 to 60 °C. As an example, Figure 5 reports the results related to the gyros biases of the MEMS IMU InvenSense MPU-9150 [29]. Ideally, such a calibration should be performed on each MEMS IMU, as they have their own characteristic curve. However, according to our experience and considering the objective of this work, the calibration performed on a single MEMS IMU is also representative of other samples of the same type and manufacturer.

A similar procedure was followed for the accelerometers. The data collected during the characterization test were gathered into a look-up table that, in turn, was used to correct the IMU measurements.

#### 2.3.2. Gyros Recalibration

The gyros bias can be estimated leaving the IMU in a static condition for a short period of time. In our design, we dedicated less than 1 s to the gyro calibration. Assuming the tightly-coupled algorithm is implemented on board of a system used for road navigation, the algorithm was designed to run a new gyros calibration any time the vehicle stops.

#### 2.3.3. Static Condition Detection

In order to detect when the vehicle stops, we implemented a simple strategy that checks the estimated velocity. When the absolute value of the velocities along the three axes is lower than a certain threshold (e.g., 0.3 m/s) we decide for the hypothesis that the vehicle is in a static condition. As long as this condition is verified, the gyro recalibration is performed. Although false detections of the static condition have been observed rarely, they can potentially occur in case of limited satellites’ visibility, as in difficult urban environments. Therefore, the proposed method could be improved with the inclusion of an additional sensor, such as an odometer.

#### 2.3.4. Nonholonomic Constraints

In case of a vehicle moving in an urban scenario, the presence of tunnels or underpasses is frequent. In such conditions, the satellite visibility is limited and the tightly-coupled algorithm can only rely on the IMU sensors to provide a navigation solution. It is well known that MEMS IMU positioning accuracy tends to diverge after a few seconds. In order to improve the performance of the algorithm in case of GNSS signals outages, NonHolonomic Constraints (NHC) were used [3]. We reasonably assume that the vertical and lateral velocities, referred to as the body frame (i.e., $V_{y}^{b},V_{z}^{b}$), are negligible and close to zero. However, it is important to highlight that such constraints can be considered a valid technique to reduce the INS drift only in case of short outage, while in the case of long outages (e.g., long tunnels or indoor parking) additional sensors are needed (e.g., odometer).

#### 2.3.5. Vertical Velocity Constraint

Since the vehicle moves on the ground, the vertical velocity can be assumed negligible. Therefore, we have modified the measurement model of the Kalman filter as stated in (10) by adding the information related to the vertical velocity that can be bounded to zero. The new $z_{k}$ vector is written as:

(13) $$z_{k}=[\zeta_{sat,k}-{\hat{\zeta }}_{k},0-V_{d}^{n}]$$

where $V_{d}^{n}$ is the vertical velocity calculated with respect to the local *l*-frame. The observation matrix can be expressed as in (14):

(14) $$H_{k}=\left[\begin{array}{cccc} H_{\rho ,k} & 0_{Nsat\times 3} & 0_{Nsat\times 3} & 0_{Nsat\times 8}\\ \;0_{Nsat\times 3} & H_{\rho ,k} & 0_{Nsat\times 3} & 0_{Nsat\times 8}\\ 0,\;0,\;0 & -C_{e,k}^{n}(3,1),-C_{e,k}^{n}(3,2),-C_{e,k}^{n}(3,3) & 0_{1\times 3} & 0_{1\times 8} \end{array}\right]\;\in \;{ℜ}^{2Nsat+1,17}$$

where $C_{e,k}^{n}$ is the DCM matrix with respect to the ECEF-frame to the *l*-frame.

#### 2.3.6. Raw Measurement Selection and Weights

In an urban environment, the user has often a limited visibility of the sky and it is necessary that the few satellites in view are weighted carefully in the PVT computation. A set of parameters are considered to assess the quality of the received signals and, in turn, the GNSS measurements. The signal quality relates to the satellite elevation, presence of multipath and other impairments, and is generally measured with the Carrier to Noise density ration (C/N_0_). Since the presence of multipath is more likely to affect satellites with low elevations, our tightly-coupled algorithm adds two masks to exclude satellites with elevations lower than 10° and a satellite showing C/N_0_ lower than 30 dB-Hz. Therefore, the algorithm is based on a model for the covariance matrix of the code-based measured pseudoranges, as proposed in [1] and [31] specifically for harsh environments:

(15) $$\sigma_{\rho }^{2}=a+b\cdot 10^{-\frac{C}{\frac{N_{0}}{10}}}$$

where $\sigma_{\rho }^{2}$ is the variance on the pseudorange estimates, a and b are empirical parameters, that are set equal to a=1 and $b=281^{2}$ for semi-urban/urban scenarios according to [1]. Our model includes also the satellite elevation, thus the formula in (15) is simply rewritten as:

(16) $$\sigma_{\rho }^{2}=\frac{\left(a+b\cdot 10^{-\frac{C}{\frac{N_{0}}{10}}}\right)}{\sin\left(Elev\right)}$$

### 2.4. Insights on Practical Implementations

The described algorithm was implemented on an embedded system. Since the objective of this paper is on the comparison of the algorithms’ performances, we keep the description of the software implementation terse. Even if the equations behind the tightly-coupled scheme do not pose severe complexity constraints, some aspects need to be carefully considered in practical implementations.

The first issue involves the synchronization between measurements. As well explained in [32], in principle low-cost GNSS receivers, MEMS IMU and other sensors generate asynchronous data. We developed a synchronization module that is used to tag the measurements from the GNSS receiver and from the IMU with respect to the same time scale. Such a module includes a time counter that counts the number of periods of the processing unit internal clock between two consecutive 1PPS signals. When a new measurement arrives, it is tagged to the value of the counter. To avoid drifts, the counter is reset at the arrival of a new 1 PPS pulse [33].

The second important aspect we consider in our implementation refers to the INS mechanization equations for real-time applications. Even if most of the microcontrollers have remarkably enhanced their computational capability, it is still important to reduce the number of operations involved in the execution of the tightly-coupled algorithm. According to [34,35], an efficient algorithm for a strapdown inertial navigation system is based on the splitting of the computing processes into low and high-speed segments. The first part is designed to take into account low frequency, large amplitude, body motions arising from the vehicle maneuvers, whereas the second involves a relatively simple algorithm that is designed to keep track of high frequency, low amplitude, motions of the vehicle. We followed this approach by choosing a computation rate for the high-speed part equal to 100 Hz, and 10 Hz for the low-speed one.

The third aspect to consider in the design of the real-time system is the management of the GNSS data latency. Every time we use a GNSS receiver, we get observables delayed with respect to the 1 PPS signal. This is due to the latency of the data output interface, to the type (and quality) of the micro-processor and its clock speed. Eventually, additional delay refers to the type of measurements to be processed (e.g., differential code/carrier-phase based observables, multi-antenna GNSS receiver for precise attitude estimation, etc.). In order to manage the GNSS data latency, INS measurements are buffered and processed only at proper time instants. For example, if a set of new GNSS measurements is expected, because the PPS signal has been received, but not yet the data message, important variables such as the estimated position, velocity, attitude, the transition and covariance matrices of the Kalman filter as well as the INS data are saved in memory, while the navigation solution is provided through the IMU measurements only. Only when the new data message is available, the status of the Kalman filter is updated and the buffers emptied.

## 3. Performance Assessment in Real Urban Scenarios

**Figure 6:**
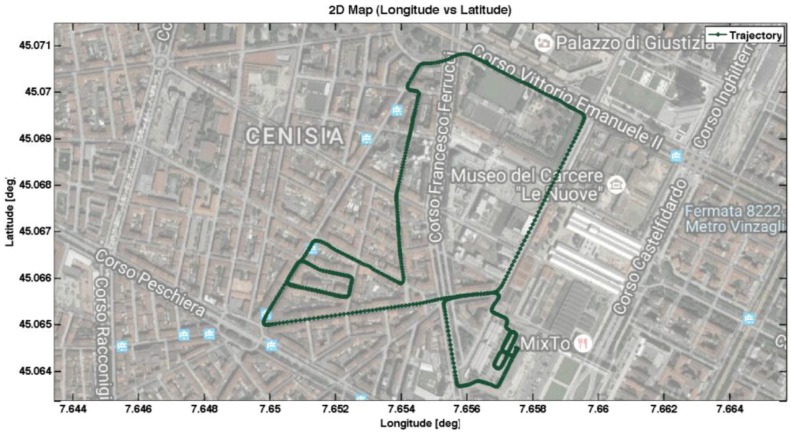
Trajectory followed during the experimental tests in Turin.

This section describes the urban scenarios selected for the experimental tests and presents the metrics used to assess the algorithms’ performances. The designed tightly-coupled architecture was implemented on an embedded system that was installed on a vehicle and used for several data collections along the trajectory shown in Figure 6 in downtown Turin.

Three portions were selected, as considered more relevant for the objective of the tests:

- Zone 1, that is a car parking area, characterized by good visibility of the sky;
- Zone 2, that is characterized by narrow streets and densely packed buildings, limiting the number of satellites in view. Moreover, in that part of the trajectory, the vehicle is expected to experience frequent stops and sharp turns;
- Zone 3, that is a straight avenue of trees, surrounded by buildings that likely generate multipath degrading the received GNSS signals.

**Figure 7:**
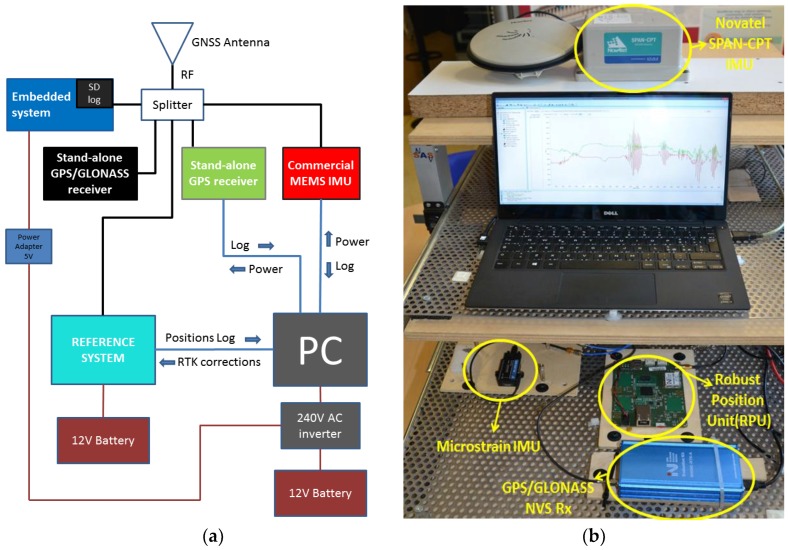
Setup (**a**) and the setup mounted on board mounted on board of a car (**b**).

In addition to the embedded system running the tightly-coupled algorithm, the experimental setup included other COTS GNSS receivers and a commercial MEMS IMU, loosely-coupled with a mass-market GPS receiver. As reference, we used a tactical-grade IMU that is tightly-coupled with a “survey-grade,” dual frequency GNSS receiver (i.e., Novatel SPAN-CPT [36]) able to provide Real Time Kinematic (RTK) positioning. When such a receiver is set to work in the RTK mode, its accuracy, in terms of RMSE, is about 2 cm along the horizontal plane and 3 cm along the vertical axis, respectively. The block diagram and the picture of the experimental setup is reported in Figure 7.

To be more specific, the principal components mounted on the embedded system running the tightly-coupled algorithm were:

- A low-cost 9-axis MEMS IMU, manufactured by InvenSense [29];
- A commercial GNSS module [37];
- A 720 MHz micro-controller Cortex-A8, running QNX as the operating system.

As mentioned, we compared our solution against two commercial standalone receivers (see the black and green blocks in Figure 7a and Table 1 for details) of the same type as that used in the embedded system. The first was configured to use only GPS satellites, the second to use both GPS and GLONASS. Last, the commercial MEMS IMU (red block in Figure 7a) was the Microstrain 3DM-Gx3-45 [23] that provided a loosely-coupled GPS/INS integration by using the embedded U-blox GPS receiver. More details on the cost and performance of all IMUs used in the setup are reported in Appendix A. Note that all the devices being tested, including the reference system, received GNSS signals from the same antenna that was the “survey-grade” AeroAntenna AT1675-382 antenna. Indeed, the quality of the antenna influences the positioning performance, but the main objective of this work was the comparison of loose and tight integrations under the same signal conditions, assessed against a reliable reference. Nevertheless, recognizing the importance of the antenna in practical operations, we also carried out additional tests using low-cost patch antennas, but the results are not reported here, because they do not provide additional insights on the comparison between loose and tight integrations.

The positioning performance was assessed through specific metrics. For all three zones, we analysed the number of satellites in view and the Horizontal Positioning Accuracy (HPA). Following the test procedures defined in the ETSI standard [38], the metric used to characterize the HPA was the horizontal position error over a specified time interval, in terms of its mean, standard deviation and 95th percentile. Furthermore, we compared the yaw angle, the Along-Track (AT) and Cross-Track (CT) errors estimated by the embedded system and the same parameters provided by the commercial Microstrain MEMS IMU [23] in the list.

For the sake of clarity, Table 1 summarizes the hardware components depicted in Figure 7a and their navigation techniques. Each configuration has been associated with a label that is then used in the following sections to comment on the results.

## 4. Results of Field Tests

This section shows the results of the test campaign, dividing the analysis among the three zones of interest. The performance of the devices being tested is compared in terms of the metrics mentioned above.

### 4.1. Zone 1: Car Parking Area

**Figure 8:**
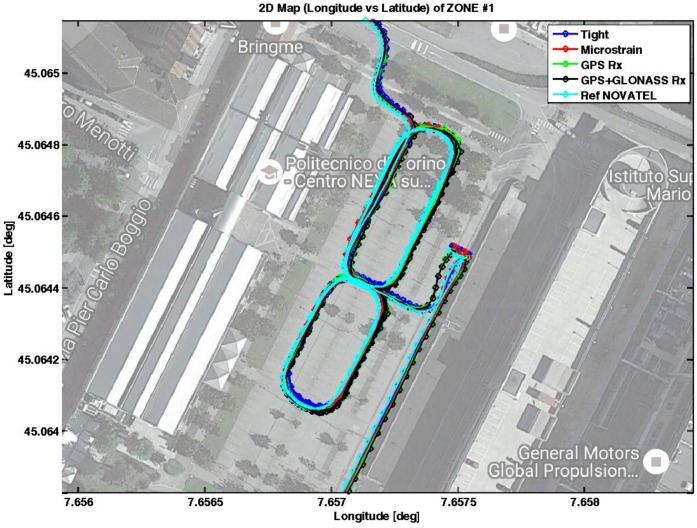
Trajectories collected by the devices being tested in Zone 1.

A zoomed view of this area is reported in Figure 8, where the trajectories recorded by the devices are reported on the map with different colors.

**Figure 9:**
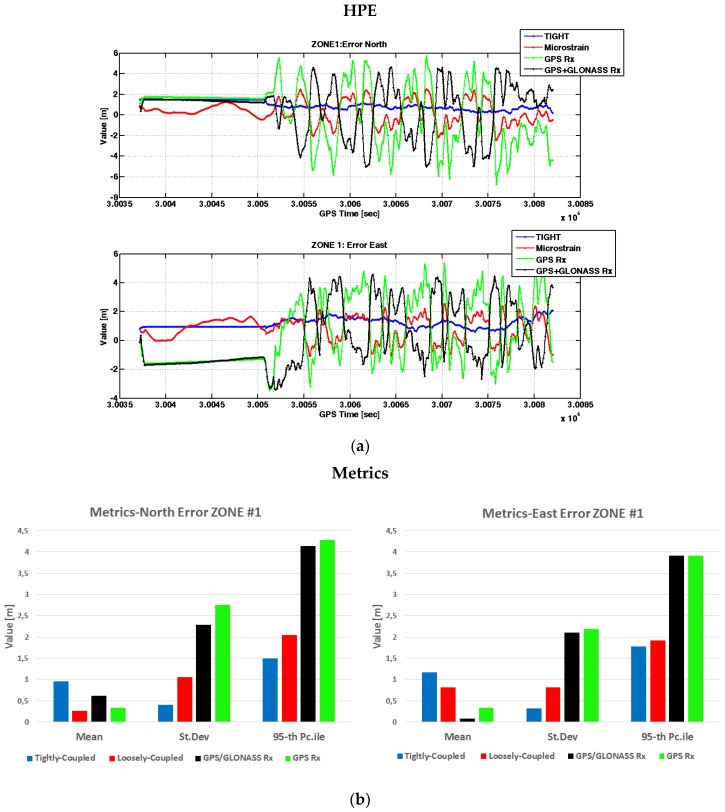
Horizontal positioning errors in Zone 1. In (**a**) measurements over time: error in the North direction (above) and error in the East direction (below). In (**b**) metrics associated to the error in the North direction (left) and to the error in the East direction (right).

The vehicle was driven in the car park, forming several figure eights. It is worth noting that the vehicle remained in a static condition for approximately 2 min before moving. On average, the number of satellites used by the multi-constellation standalone receiver (black curve) was 16, while all other devices, configured to process only GPS signals, received 10 satellites. The advantage of having more satellites in view is more evident in the other zones, where the number of satellites is sometimes not sufficient to provide positions, if the receiver relies only on GPS. The horizontal positioning errors are plotted in Figure 9 on the North and East coordinates. Note that such errors are computed by subtracting the coordinates estimated by the devices under investigation and those provided by the reference receiver, at the same time instants.

From Figure 9, it is possible to observe how the tightly-coupled algorithm (blue lines) provides the best performance in terms of precise estimates of the vehicle position. The tight integration shows the lowest standard deviations and 95th percentiles of the error on both the coordinates. Although a small bias approximately equal to 1 m affects the position accuracy of the tight solution, we observe the effect of the static condition constraints in the first part of the data collection, which maintains the estimated position constant while the vehicle is actually still. Different reasons could have generated the presence of bias in the positional solution obtained through the designed tightly-coupled technique. Most likely, the reference system used a higher number of satellites to compute the PVT with respect to our solution and had a lower dilution of precision. In addition, the reference system was set to work in RTK mode, exploiting differential carrier-phase measurements, that allows for achieving an accuracy of 2 cm. On the contrary, our solution was based only on code-based pseudoranges. The commercial MEMS IMU (red lines) has an error that varies from −2 to 2 m both in the North and East axes, while the standalone GPS receiver shows errors ranging from −5 to 5 m along the North coordinate and from −3 to 4 along the East coordinate, respectively. The benefits of multi-constellation cannot be noted in this scenario due to the high number of GPS satellites in view. Indeed, as evident from the same level of standard deviations and 95th percentiles, the performance of the GPS/GLONASS receiver (black columns in Figure 9b) is comparable with that obtained by the GPS receiver (green columns in Figure 9b).

**Figure 10:**
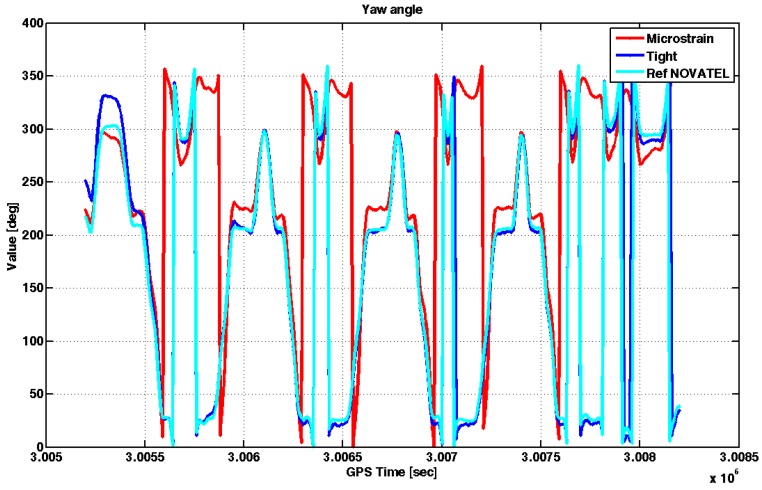
Yaw angles estimated by different MEMS IMU sensors under investigation in the Zone 1.

Figure 10 shows the yaw angles estimated by the embedded system running the tightly-coupled algorithm (blue line) and those obtained by the commercial MEMS IMU (red line). The figure also plots in cyan the yaw angles estimated by the reference receiver.

**Figure 11:**
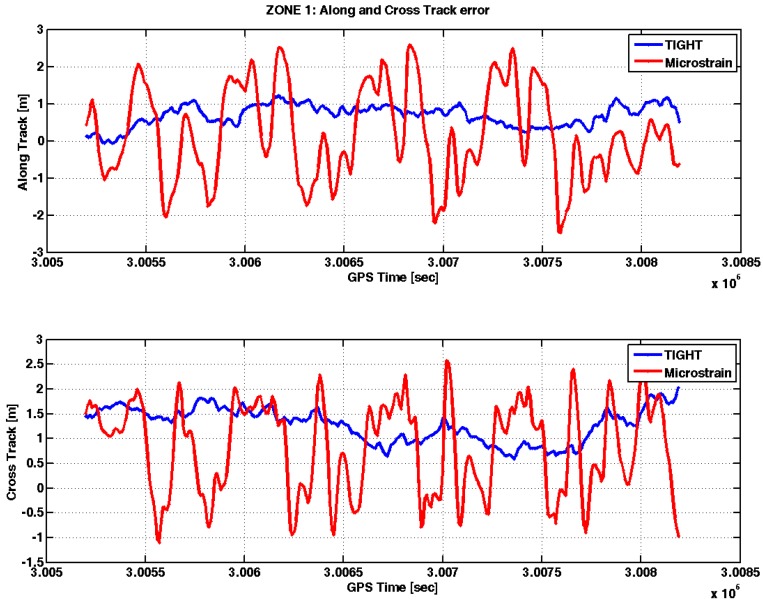
Along-Track (AT) and Cross-Track (CT) errors in Zone 1.

In Figure 10 the initial part in static has been omitted since the reference receiver provided a valid attitude value only in dynamic. The tightly-coupled algorithm needs tens of seconds to converge to the right solution: such behavior is due to the low-cost IMU used in the embedded system that is not able to provide a valid initial heading angle due to the high level of noise affecting its gyroscopes. However, after this transient, the yaw angle estimated by our system is comparable to that of the reference receiver, as the corresponding curves in Figure 10 are almost superimposed. Conversely, the quality of the measurements provided by the commercial MEMS IMU seems poor, as the difference with respect to the reference is on the order of tens of degrees, even in open sky conditions. Eventually, also the AT and CT errors have been calculated and can be seen in Figure 11.

Considering the AT error, in open sky conditions, the tightly-coupled algorithm is able to bound it within 1 m, while the commercial MEMS IMU shows an error ranging from −2 to 2 m. On the other hand, Figure 11 confirms what was commented on above, that the 1 m bias affects the estimated positions also in the AT-CT frame by using the tightly-coupled algorithm. The commercial MEMS IMU shows a much smaller bias, although the position estimates are less precise. The details of the loosely-coupled algorithm implemented in the commercial MEMS IMU are not known. Similar to the offset experienced by our tightly-coupled algorithm, such a small bias is likely due to the use of code-based pseudorange measurements and to a lower number of satellites used in the PVT. Moreover, the bias experienced with the Microstrain is different from the bias obtained with the tightly-coupled algorithm, because of different strategies in the selection and weight of the satellite measurements included in the PVT computation.

### 4.2. Zone 2: Urban Canyon

**Figure 12:**
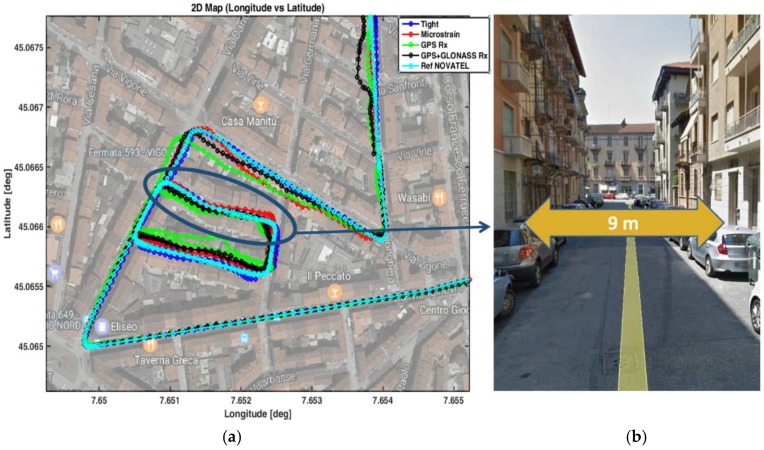
Trajectories collected by the devices being tested in Zone 2 (**a**) and picture of a narrow street passed through during the test (**b**).

The left part of Figure 12 shows the trajectories recorded by the devices being tested in Zone 2 that is characterized by narrow streets, tightly packed buildings and reduced sky visibility. A snapshot of the path is visible on the right side of Figure 12 with a zoomed view, taken from Google Earth.

In such a challenging scenario, the number of satellites in view plays a fundamental role in obtaining accurate navigation performance. The number of GPS satellites is remarkably reduced with respect to Zone 1. Although the multi-constellation standalone receiver (black curve) still guarantees at least 12 satellites in view most of time, the tightly-coupled algorithm worked, on average, with 5−6 satellites. The reference system tracked also satellites with degraded C/N_0_ and had a higher number of GPS satellites in tracking with respect to the mass market GPS receiver used by the embedded system.

**Figure 13:**
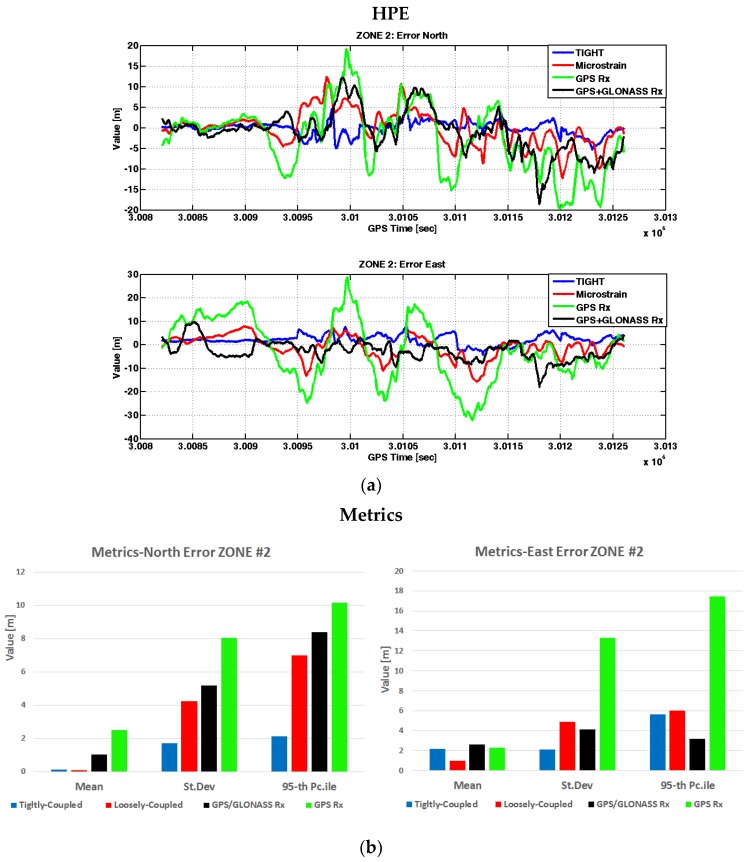
Horizontal positioning errors in Zone 2. In (**a**) measurements over time: error in the North direction (above) and error in the East direction (below). In (**b**) metrics associated to the error in the North direction (left) and to the error in the East direction (right).

Similar to Zone 1, the horizontal positioning errors are plotted in Figure 13 on the North and East coordinates, for all the devices under investigation.

From Figure 13 we appreciate the benefits of the tightly-coupled algorithm (blue lines) with respect to the loosely-coupled one (red lines). The tight strategy provides accurate position estimates, with the lowest standard deviations (i.e., 1.69 m on the North and 2.10 m on the East) and the 95th percentiles (i.e., 2.13 m on the North and 5.60 m on the East) of the errors. In this test case, the embedded system running the tightly-coupled algorithm outperformed the commercial MEMS IMU. The horizontal position error was always lower than 5 m along the North coordinate, and lower than 8 m along the East coordinate. As expected, there is a general degradation of the positioning performance of all devices, passing from Zone 1 to Zone 2. Clearly, the standalone GPS receiver (green lines) does not offer similar performance and showed errors up to 30 m, with a standard deviation of the error on the order of ten meters. Furthermore, considering the measurements from the two un-coupled GNSS receivers, we notice the benefits brought by multiple constellations that results in improved positioning performance when the visibility of the satellites belonging to one constellation is reduced. The multi-constellation receiver (black lines) has significantly better performance with respect to the single constellation receiver, even comparable with the ones obtained by the commercial MEMS IMU.

**Figure 14:**
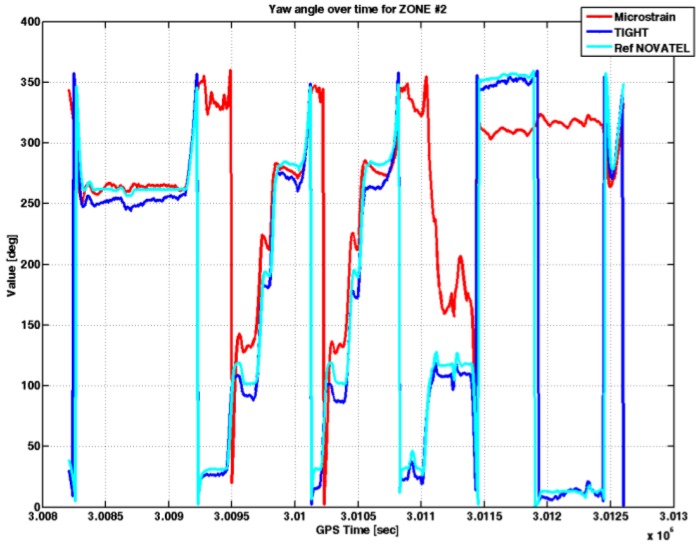
Yaw angles estimated by different MEMS IMU sensors under investigation in Zone 2.

Figure 14 shows the yaw angle estimated by the embedded system running the tightly-coupled algorithm and the commercial MEMS IMU.

**Figure 15:**
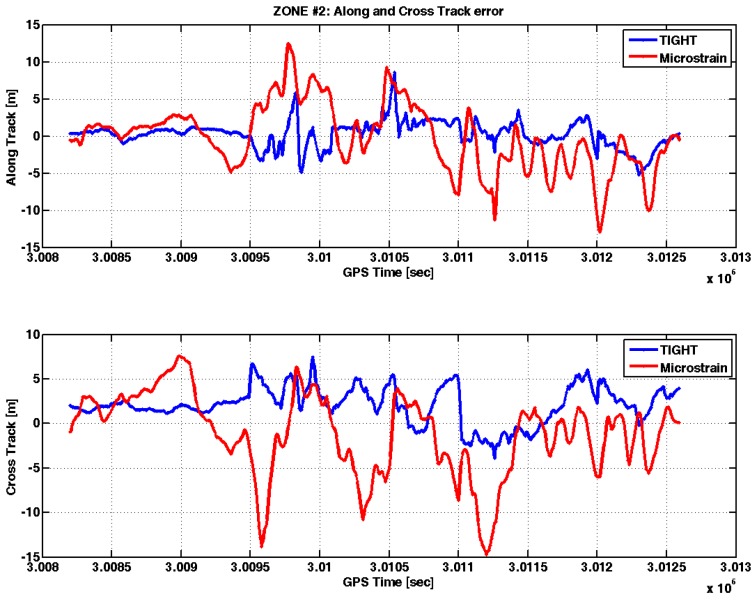
AT and CT errors in Zone 2.

In Zone 2, the yaw angle estimated by the embedded system (blue line) followed the trend of the yaw angle estimated by the reference (cyan line). In certain time instants, it is possible to note a difference that reaches values up to 20°. However, the accuracy of the yaw angle, as calculated by the tightly-coupled strategy, is much better with respect to that provided by the commercial MEMS IMU. In fact, the standard deviation of the error on the estimated yaw angle is 5.28° using the embedded system, against 44.3° measured on the angles estimated by the commercial MEMS IMU. The AT and CT errors over time are shown in Figure 15.

The AT and AC errors are lower with the tightly-coupled algorithm (blue line) with respect to the loosely-coupled approach used by the commercial MEMS IMU (red line). In this test case, the standard deviation of the AT and CT errors are approximately 1.8 m and 2.06 m, against 4.5 m and 4.6 m experienced with the commercial MEMS IMU. The reason is twofold: first, the tight integration allows for more degrees of freedom in the design of the integration Kalman filter; second, the better accuracy of the estimated yaw angles is certainly an advantage to obtain small AT and CT errors.

### 4.3. Zone 3: Straight Avenue of Trees

**Figure 16:**
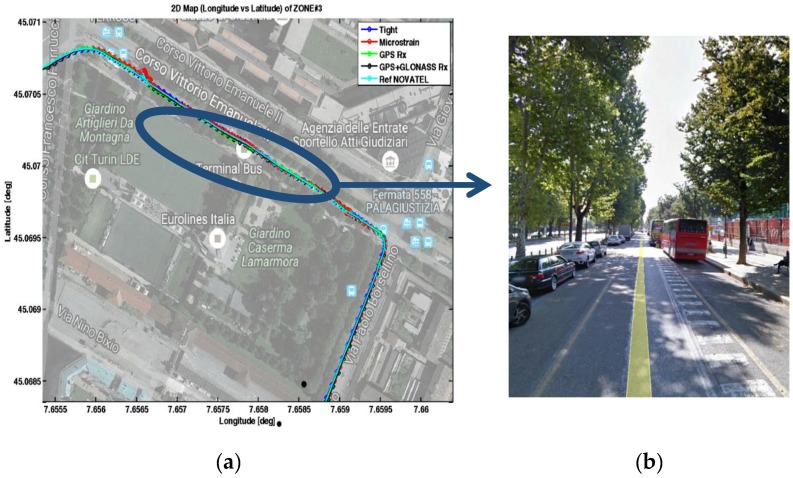
Trajectories collected by the devices being tested in Zone 3 (**a**). A tree-lined street is depicted in (**b**).

Figure 16 shows the trajectories recorder during the data collection performed along a tree-lined avenue. During the test, the vehicle was stopped under the trees for 165 s to better validate the performance of the algorithm with limited satellite view and degraded received signal. Figure 16 also shows the picture of the tree-lined avenue, taken from Google Earth.

Compared to Zone 2, this scenario appears less critical. Indeed, the GNSS receiver used by the embedded system tracks on average eight satellites. Obviously, the multi-constellation receiver is able to track the highest number of satellites: in this case, it is 15 most of the time. Only in a few instants does the number of satellites drop to 14.

**Figure 17:**
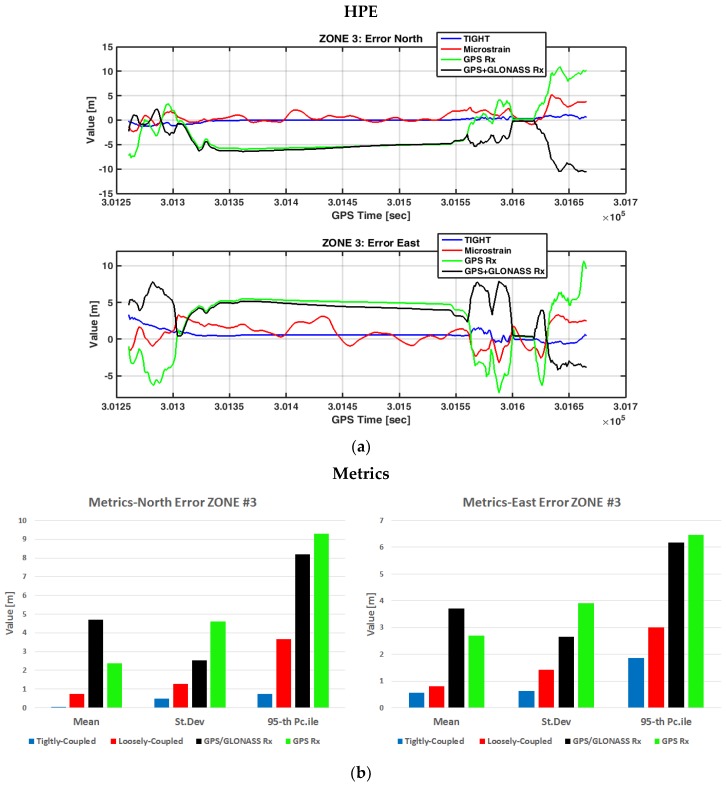
Horizontal positioning errors in Zone 3. In (**a**) measurements over time: error in the North direction (above) and error in the East direction (below). In (**b**) metrics associated to the error in the North direction (left) and to the error in the East direction (right).

The horizontal positioning error over time is plotted in Figure 17, for all the devices being tested.

Also in this scenario, the tightly-coupled algorithm provided the lowest error with respect to all the other sensors used in the test. We notice that the standard deviation of the horizontal errors is lower than 1 m on both the North and East coordinates, whereas the 95th percentiles do not reach 2 m. The results of this test case are similar to those obtained in open sky conditions. This proves the robustness of the tight integration that is able to provide reliable position estimates also in conditions of low satellite visibility. Also in this zone, the embedded system outperformed the commercial MEMS IMU (red lines). However, from this test, it is possible to observe the advantages brought by the GNSS/INS integration, either following a loosely-coupled (red lines) or a tightly-coupled (blue lines) architecture. Both showed superior performance with respect to standalone receivers, both single (green lines) and multi-constellation (black lines). In particular, the designed tightly-coupled strategy maintains a small standard deviation on the estimated positions and, thanks to the velocity constraints, it maintains the solution constant the whole time the car is static. In this test case, the two standalone receivers showed similar performance. Contrary to Zone 2, where sometimes the number of GPS satellites was not sufficient to compute the user’s position, in this scenario the advantage of the multi-constellation cannot be appreciated. Indeed, during the test, the number of tracked GPS satellites never decreased below six and the dilution of precision remained below 1.2.

**Figure 18:**
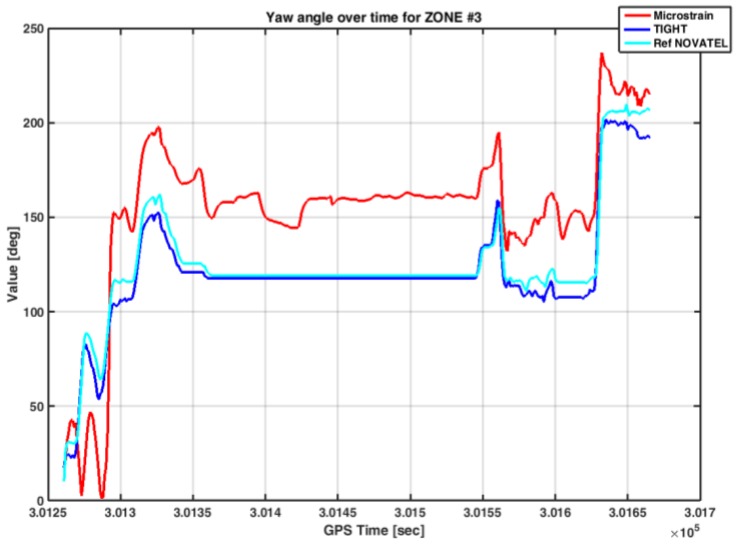
Yaw angles estimated by different MEMS IMU sensors under investigation in Zone 3.

Figure 18 shows the yaw angle estimated by the embedded system running the tightly-coupled algorithm and the commercial MEMS IMU.

The error of the estimated yaw angle is quite large for the case of the loosely-coupled algorithm implemented in the commercial MEMS IMU. Such a poor quality of the yaw estimate is in line with the performance experienced in the other zones.

On the contrary, the designed tightly-coupled algorithm provided a yaw angle similar to that estimated by the reference, even if in some time intervals (e.g., GPS time from 301,290 to 301,315 and from 301,600 to 301,635) the difference between them was up to 15°. Conversely, we observe how the yaw angle is maintained constant during the long static conditions experienced by the vehicle.

**Figure 19:**
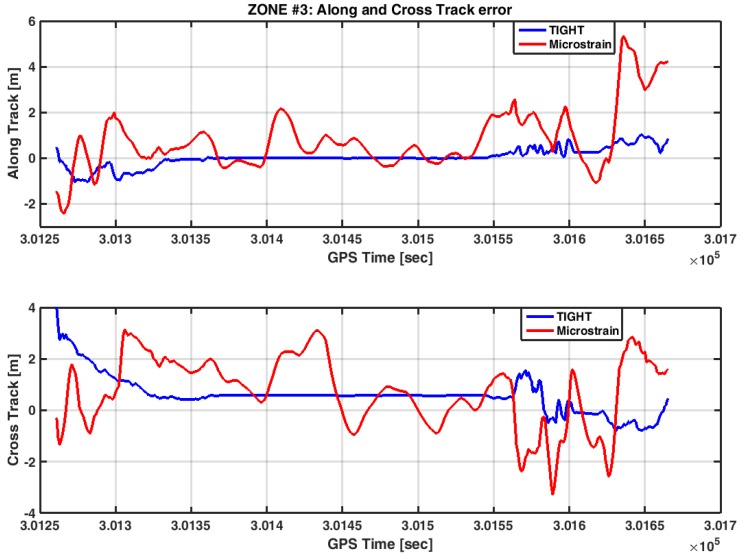
AT and CT errors in Zone 3.

Eventually, Figure 19 reports the AT and CT errors computed in Zone 3.

The loosely-coupled strategy provides positions in the AT and CT framework, with errors to the order of few meters. The standard deviation of the AT and CT error is higher if compared to the one experienced by the designed tightly-coupled algorithm. Similar to Zone 2, the poor estimate of the yaw angles affects the positioning performance in the AT-CT frame. In Appendix B we have summarized the results obtained in each scenario, for all the receivers being tested.

## 5. Conclusions

This paper presents the assessment of the positioning performance of a Global Navigation Satellite Systems (GNSS)/Inertial Navigation Systems (INS) tightly-coupled algorithm, measured in real urban scenarios. The algorithm was designed to fuse measurements from a low-cost INS and a mass-market Global Positioning System (GPS) receiver. Results show a significant decrement of the positioning errors, if compared to those obtained with other commercial devices. In particular, the tightly-coupled algorithm provides better estimates of the vehicle position and attitude, with respect to a commercial GPS module, loosely integrated with an inertial sensor. The improvement was measured following a standardized testing method, considering the horizontal position error and the yaw angle, as the main performance metrics. The experimental results reported in this paper demonstrate the possibility to employ tightly-coupled architectures also in mass-market devices, often employed in applications where users move in urban spaces. Examples include pay-as-you-drive insurances, tracking of fleet for winter road maintenance, systems for advanced driver assistance and autonomous vehicles. In the years ahead, the improvement of Micro Electrical Mechanical Sensors (MEMS) technology and the evolution of GNSS, with enhanced signal formats, different frequency bands and more satellites in view, are expected to further increase the positioning performance of mass-market devices, enabling a variety of new services for road users.

## Figures and Tables

**Table 1:** Hardware components and navigation technique used during the tests.

Label	Hardware	Navigation Technique
Tight	Embedded System	GPS+INS tightly-coupled algorithm
Microstrain	Commercial MEMS IMU (i.e., Microstrain 3DM-Gx3-45)	GPS+INS loosely-coupled algorithm
GPS Rx	Standalone GPS receiver (i.e., NVS NV08C-CSM)	Standalone GPS PVT solution
GPS+GLONASS Rx	Standalone GPS/GLONASS receiver (i.e., NVS NV08C-CSM)	Standalone GPS+GLONASS PVT solution
Ref NOVATEL	REFERENCE SYSTEM (i.e., Novatel SPAN-CPT)	Dual Frequency GPS, RTK+INS tightly-coupled algorithm

## Appendix A

In Table A1 we have also summarized the main features and the price of the IMU sensors that have been used during the test in the urban area. Only the gyros characteristics have been reported because they represent the most important error sources in case of MEMS IMU. In fact, the gyro error has a direct influence on the attitude accuracy since the GNSS Kalman filter updates can only directly compensate for the position and velocity error while the attitude error relies mostly on the IMU calibration and the proper gyro bias correction.

**Table A1:** A selection of sensor error distributions derived from their datasheets, in the units given. The InvenSense is a consumer-grade MEMS. The Microstrain is a factory-calibrated MEMS IMU and the Novatel is a tactical-grade IMU.

Sensor Manufacturer	InvenSense	Microstrain	Novatel
Model	MPU-9150	3DM-Gx3-45	SPAN-CPT
Type	Single-chip IMU	Factory calibrated IMU	Factory calibrated IMU
Price	<100$	3000$	25000$
Gyroscope Errors			
Bias Offset	±20 dps *	±0.25 dps	±0.0056 dps
In-Run Stability	Not Specified	18°/h	± 1°/h
Noise	0.005 dps/$\sqrt{Hz}$	0.03 dps/$\sqrt{Hz}$	1.86 × 10^–5^ dps/$\sqrt{Hz}$
Scale Factor Errors	±3%	±0.05%	±0.0015%
Non-Linenearity	±0.2% FS ^†^	±0.03%	Not Specified
Cross-Axis Sensitivity	±2% FS	Aligned to ±0.05°	Not Specified

* dps = degree per second, ^†^ FS = Full Scale.

## Appendix B

**Table A2:** Summary of performance of the receivers being tested in Zone 1 (Open Sky).

Sensor Manufacturer		Custom-Board	Microstrain	NVS	NVS
Model		MPU-9150	3DM-Gx3-45	NV08C-CSM	NV08C-CSM
Nav Algorithm		Tightly-coupled	Loosely-coupled	GPS/GLONASS	GPS
	Mean, St. Dev, 95th PCTL				
		0.95	0.27	0.62	0.33
Position North [m]		0.41	1.05	2.28	2.75
		1.5	2.05	4.14	4.28
		1.17	0.81	0.08	0.33
Position East [m]		0.32	0.82	2.10	2.19
		1.78	1.92	3.91	3.91
		1.46	14.43	N.A. *	N.A.
Heading [deg]		9.04	27.63	N.A.	N.A.
		8.11	56.3	N.A.	N.A
Along Track [m]		0.29	1.24	N.A.	N.A.
		1.08	2.15	N.A.	N.A.
		1.28	0.80	N.A.	N.A.
Cross Track [m]		0.35	0.92	N.A.	N.A.
		1.79	2.03	N.A.	N.A.

N.A.* = Not Available.

**Table A3:** Summary of performance of the receivers being tested in Zone 2 (Urban Canyon).

Sensor Manufacturer		Custom-Board	Microstrain	NVS	NVS
Model		MPU-9150	3DM-Gx3-45	NV08C-CSM	NV08C-CSM
Nav Algorithm		Tightly-coupled	Loosely-coupled	GPS/GLONASS	GPS
	**Mean, St. Dev, 95th PTCL**				
		0.12	0.1	1.02	2.50
Position North [m]		1.69	4.23	5.19	8.04
		2.13	6.98	8.39	10.16
		2.14	0.96	2.60	2.27
Position East [m]		2.10	4.85	4.10	13.29
		5.60	6.00	3.15	17.43
		8.90	19.08	N.A.*	N.A.
Heading [deg]		5.28	44.33	N.A.	N.A.
		18.66	68.05	N.A.	N.A
		0.22	0.25	N.A.	N.A.
Along Track [m]		1.80	4.47	N.A.	N.A.
		2.6	7.15	N.A.	N.A.
		2.08	1.16	N.A.	N.A.
Cross Track [m]		2.06	4.58	N.A.	N.A.
		5.24	5.51	N.A.	N.A.

N.A.* = Not Available.

**Table A4:** Summary of performance of the receivers being tested in Zone 3 (Avenue of trees).

Sensor Manufacturer		Custom-Board	Microstrain	NVS	NVS
Model		MPU-9150	3DM-Gx3-45	NV08C-CSM	NV08C-CSM
Nav Algorithm		Tightly-coupled	Loosely-coupled	GPS/GLONASS	GPS
	**Mean, St. Dev, 95th PTCL**				
		0.1	0.8	4.64	2.45
Position North [m]		0.54	1.13	2.59	4.85
		0.83	3. 8	8.1	9.2
		0.64	0.86	3.70	2.87
Position East [m]		0.82	1.45	2.80	3.97
		1.91	1.90	6.12	6.53
		4.38	40.4	N.A. *	N.A.
Heading [deg]		3.87	53.69	N.A.	N.A.
		11.28	65.34	N.A.	N.A
		0.02	0.79	N.A.	N.A.
Along Track [m]		038	1.32	N.A.	N.A.
		0.6	3.85	N.A.	N.A.
		0.58	0.72	N.A.	N.A.
Cross Track [m]		0.69	1.38	N.A.	N.A.
		2.04	2.82	N.A.	N.A.

N.A.* = Not Available.
